# A Retained Bullet in Pericardial Sac: Penetrating Gunshot Injury of the Heart

**DOI:** 10.1155/2016/2427681

**Published:** 2016-02-10

**Authors:** Adnan Kaya, Emine Caliskan, Mustafa Adem Tatlisu, Mert Ilker Hayiroglu, Ahmet Ilker Tekessin, Yasin Cakilli, Sahin Avsar, Ahmet Oz, Osman Uzman

**Affiliations:** ^1^Cardiology, Suruc State Hospital, Sanliurfa, Turkey; ^2^Radiology, Suruc State Hospital, Sanliurfa, Turkey; ^3^Cardiology, Dr. Siyami Ersek Cardiovascular and Thoracic Surgery Hospital, Istanbul, Turkey

## Abstract

Penetrating cardiac trauma is rarely seen but when present there is a short time lag to keep the patients alive. Cardiac gunshot injuries are exceptional and it occurs mostly during interpersonal disagreements casualties or a mistakenly fired gun nowadays. Here we present a case of cardiac gunshot injury from the war of Kobani, Syria. The patient was mistakenly diagnosed to have a sole bullet in the left shoulder while he had a penetrating cardiac trauma with a bullet in the heart and pericardial effusion possibly giving rise to pericardial tamponade. Luckily the cardiac gunshot injury was noticed one day later and the patient was referred to a tertiary hospital. Intrapericardial bullet was conservatively followed up. The patient was discharged one week later after resection of the bullet in the shoulder.

## 1. Introduction

Penetrating cardiac trauma is rarely seen but when present there is a short time lag to keep the patients alive. Cardiac gunshot injuries are exceptional and they occur mostly during interpersonal disagreements casualties or a mistakenly fired gun nowadays. Most cases of the literature of cardiac gunshot injuries come from the data of World War II [1] and Lebanese civil war [2]. Nearly 81% of patients with cardiac gunshot injury lost their life [3]. When a cardiac gunshot injury is suspected computerized tomography, transthoracic echocardiography, and transesophageal echocardiography are suggested for evaluation of cardiac compromise, bullet trajectory, and the localization of the bullet [4]. Surgical intervention is gold standard when hemodynamic compromise like pericardial tamponade, hypovolemic shock due to bleeding is present. However, there is no treatment consensus of hemodynamic stable patient with cardiac gunshot injury and it varies from patient to patient.

Here we present a case of cardiac gunshot injury from the war of Kobani, Syria. The patient was mistakenly diagnosed to have a sole bullet in the left shoulder while he had a PCT with a bullet in the pericardial sac and pericardial effusion possibly giving rise to pericardial tamponade. One day later the bullet in the heart was noticed and luckily the pericardial effusion was shifted to the right pleura. With this case report we would like to point out cautious interrogation of gunshot wounds despite clinical stability at admission and follow-up.

## 2. Case Presentation

**Figure 1:**
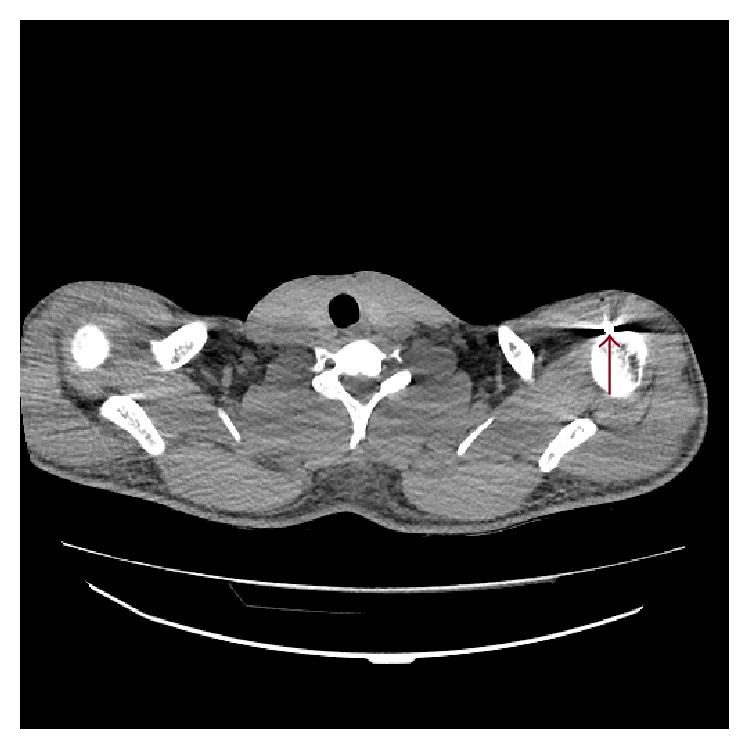
Transverse cut view of chest CT shows a bullet in the left shoulder at proximal humerus.

A 32-year-old male fighter was brought to our emergency department on the morning of 6th January, 2015, with several other wounded fighters from the war of Kobani, Syria. Because of the ongoing war, there have been many gunshot wounds, bomb blast wounds, and deaths admitted to our ED. Whenever a casualty arrived to the ED all the staff intensify for minimizing the time loss to save the patients. Fast triage for all the patients is required to determine which patients need to refer to tertiary hospitals and which patients to intervene on the scene. This patient arrived to our hospital after a suicide bomb attack with nine other patients. Physical examination revealed a superficial wound on the left shoulder and another on the left side of thorax at the 6th intercostals space. There were no exit sides of these wounds which were thought to be bullet wounds. He was conscious with a Glasgow Coma Score of 15, a blood pressure of 110/55 mmHg, a body temperature of 36.3°C, respiratory rate of 22/minute, an oxygen saturation of 93% on room air, and a heart rate of 103 beats per minute. After first evaluation of the patient a thoracoabdominal computerized tomography (CT) was ordered. A bullet was seen in the left shoulder at proximal humerus (Figure 1). There was no fracture of the bone. Intravenous saline, antibiotic, and intramuscular tetanus vaccine were started and the patient was admitted to the general surgery ward for extraction of the bullet. There was moderate dyspnea of the patient which was attributed to anxiety and pain shock. The patient was mobilized during the evening. He had severe dyspnea which was resolved suddenly at 01.00 am and he slept till 06.30 without any symptoms.

**Figure 2:**
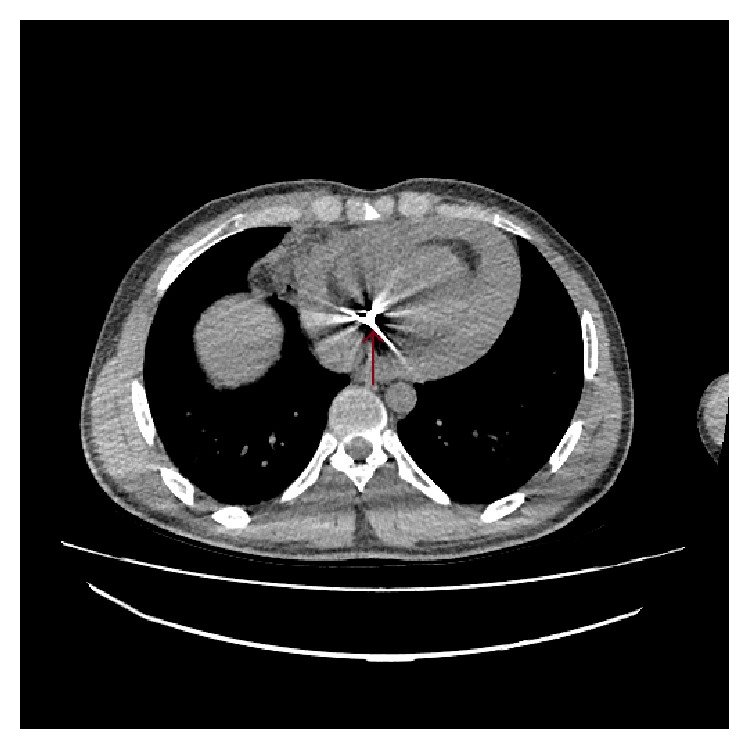
Transverse cut view of chest CT shows a bullet in the pericardial sac with pericardial effusion.

**Figure 3:**
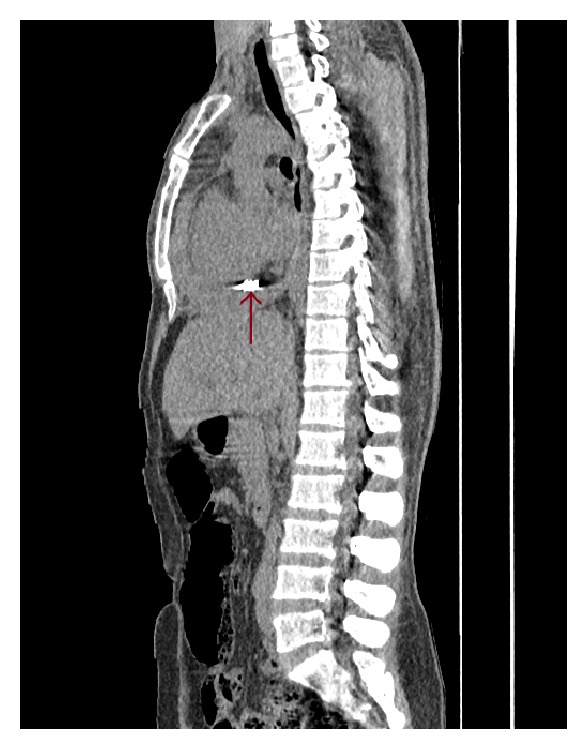
Sagittal cut view of chest CT shows a bullet in the pericardial sac with pericardial effusion.

The radiologist and the general surgeon of the hospital noticed a pericardial bullet in the patients CT while checking it. The CT shows a hyperdense material in the heart with up to 2 cm pericardial effusion. Transverse cut view of chest CT shows a bullet in the pericardial sac with pericardial effusion compressing the heart (Figure 2). Sagittal cut view of chest CT also revealed a bullet in the pericardial sac with pericardial effusion (Figure 3). There was no evident projectile trajectory.

**Figure 4:**
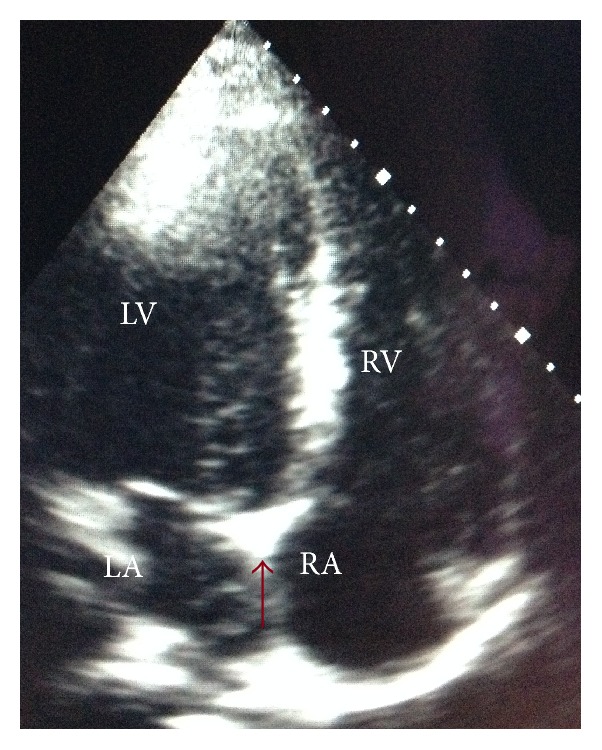
Modified apical 4-chamber view of TTE shows the bullet without pericardial effusion.

Cardiology consultation was made. A bedside physical evaluation showed normal heart sounds without any murmurs. Electrocardiography showed sinus rhythm with a rate of 67 bpm and no abnormality. A bedside transthoracic echocardiography (TTE) showed a hyperechogenicity embedded near to the connection of interatrial and interventricular septum in apical 4-chamber view (Figure 4); however, a definite conclusion could not be made if the bullet is compromising the myocardium. There were no valvular insufficiency and no interventricular or interatrial connections. The pericardial effusion seen in CT was drained to the right pleura. The patient was stable. No pericardial effusion, no valvular insufficiency, and no rhythm abnormalities were observed.

The patient was referred to a tertiary cardiovascular institution for follow-up and definite treatment. After a detailed evaluation conservative follow-up was decided and the patient was observed one week in the coronary care unit. He was discharged on the 13th of January after resection of the bullet in the left shoulder. He showed up to his first month visit without any complaints. We do not have any news from him since then.

## 3. Discussion

The most important clinical presentation of cardiac gunshot injuries is those leading to the cardiac wound, pericardial tamponade, and intrathoracic bleeding. Valvular insufficiencies intracardiac shunts, and conduction defects could be seen in the early course of the trauma. Bacterial endocarditis [5], pericarditis [6], systemic or pulmonary embolization of the missile or thrombi, and neurotic manifestations of various degrees could be late presentations.

The management of cardiac gunshot injuries depends on hemodynamic compromise of the patient. Surgical intervention and correction are an obligation when hemorrhagic shock due to blood loss and pericardial tamponade is present. Some patients may have no time to reach healthcare institution due to severe blood loss and tamponade. When a patient suffers from a cardiac gunshot injury with stable hemodynamic, a careful evaluation is warranted. A thorough physical examination with evaluation vital signs must be the first step. Then chest X-ray and chest CT must be ordered for the possible damage of the vital organs and for localization foreign material. TTE is performed to confirm pericardial effusion and localization of foreign material. Despite progress in surgical interventions and postoperative care the management of retained missiles in the heart and pericardium is still controversial [7–10].

Surgical care of gunshot wounds of the heart and the descending aorta and thoracic or abdominal aorta focuses on substantial and continuous blood loss [11]. Resuscitation management of these penetrating injuries involves massive volume replacement of colloid and crystalloid solutions as well as of blood [12]. Other organ injuries (liver, lungs, stomach, and small intestines) may worsen the situation and complicate the resuscitation. Early surgical intervention may be the only diagnostic and therapeutic procedure at hand, as rapid operative control of the hemorrhagic site is the most effective resuscitation manoeuvre [13]. Correction of damaged tissue must be performed as fast as possible. Projectile trajectory must be followed up for injured tissues. A successful surgical gunshot injury of 16-year-old boy was presented by Aydemir et al. [14]. In this case the projectile entered from right anterior thoracic cavity, passed through the right lung, right atrium, atrial septum, left ventricle, and left lung, and ended up between the eighth and ninth ribs. Early surgical intervention saved this patient's life.

In our case the first examination of the patient revealed a stable hemodynamic and two inlet wounds without exit. A CT of chest was mistakenly reported as the patient has only one bullet in the left shoulder while he had one in the myocardium with pericardial effusion. Because of our increased workload with nine other casualties no one noticed the CT finding and the patient was interned to the ward for removal of the bullet in the shoulder. The patient's dyspnea is resolved by itself suddenly and he had a comfortable sleep. TTE was performed for the pericardial effusion and intracardiac bullet. TTE showed an embedded bullet to the connection of interatrial and interventricular septum. There was no valvular compromise and electrical instability. Pericardial effusion was drained to the right pleura and the patient was stable. Resection of the bullet of the shoulder was performed and the patient was discharged without any intervention after one-week follow-up in the ward of the tertiary hospital.

## 4. Conclusion

Cardiac gunshot injuries are very rare in daily routine of medical practice. Physical examination, chest X-ray, chest CT, and TTE are the key elements for rapid diagnosis and management. Here we present a case of cardiac gunshot injury and missed diagnosis because of increased workload. By this case we would like to draw attention to a careful evaluation of gunshot wounds whatever the clinical situation of the patient is.
